# Aortic Remodeling After False Lumen Embolization in Aortic Dissection

**DOI:** 10.3390/jcm14030763

**Published:** 2025-01-24

**Authors:** Nancy Halloum, Anna-Sophie Meyer, Martin Wenkel, Daniel-Sebastian Dohle, Marwan Youssef, Bernhard Dorweiler, Hendrik Treede, Hazem El Beyrouti

**Affiliations:** 1 Department of Cardiac and Vascular Surgery, University Medical Centre Mainz, Johannes Gutenberg University, 55131 Mainz, Germany; 2 Department for Vascular Surgery, Asklepios Nord Clinic, 22417 Hamburg, Germany; 3 Department of Vascular and Endovascular Surgery, Faculty of Medicine, University Hospital of Cologne, University of Cologne, 50937 Cologne, Germany

**Keywords:** thoracic aorta, chronic dissection, TEVAR, endovascular, candy-plug

## Abstract

**Background/Objectives**: Retrograde false lumen perfusion after thoracic endovascular aortic repair (TEVAR) can compromise positive remodeling and clinical outcomes. The aim of this study is to describe the feasibility and outcomes of a false lumen thrombosis technique. **Methods**: A single-center, retrospective analysis of patients between January 2017 and January 2022 who underwent TEVAR in conjunction with false lumen embolization. **Results**: Twelve patients (83% type A, 17% type B; 33% with frank rupture; mean age 65 years; eleven men) underwent 13 false lumen embolization procedures with a 92% technical success rate and a 100% clinical success rate. Positive aortic remodeling was observed in all the patients. The maximum thoracic aortic diameter remained stable (*p* = 0.526) but the true lumen increased from 22 to 33 mm (*p* = 0.009) and the false lumen decreased from 41 to 20 mm (*p* = 0.076) after a median follow-up of 31 months. **Conclusions**: False lumen embolization using the candy-plug is safe and promotes positive aortic remodeling.

## 1. Introduction

Aortic dissection is associated with a significant mortality rate if not addressed; specifically, type A aortic dissection (TAAD) can exhibit a 30-day mortality rate as high as 90% [1]. Research suggests a rising prevalence of acute TAAD, with annual estimates varying between 4.7 and 13.6 cases per 100,000 individuals [2,3]. Over the last few years, thoracic endovascular aortic repair (TEVAR) has emerged as one of the most frequently performed surgical procedures for various thoracic aortic conditions [4].

Although thoracic endovascular aortic repair (TEVAR) can adequately seal the primary entry tear in aortic dissections, pressure in the false lumen can persist due to retrograde flow through distal re-entry tears [5]. Persistent false lumen perfusion is associated with a risk of rupture and is predictive of aneurysmal degeneration and poor outcomes [6]. In chronic aortic dissections, the dissecting lamella increasingly loses flexibility and distal re-entry tears become stable communications between the true and false lumens; partial thrombosis of the false lumen may occur over time but is often limited to stented segments [7]. In addition, preoperative thoracic false lumen branches are a morphologic predictor of thoracic aortic enlargement and may be an indication for the need for false lumen embolization [8].

Coil embolization, as well as knickerbocker and candy-plug stent graft designs, have been used to achieve false lumen thrombosis, with a reduction in and positive remodeling of the dissected aorta. The main difference between the knickerbocker and candy-plug techniques is the level at which occlusion can be achieved; this is more proximally with the knickerbocker, more distally with the candy-plug [9]. Another method reports the diversion of the false lumen flow to a nearby branch vessel [10]. With the candy-plug technique, the device should be positioned into the false lumen distally aligned to a true lumen stent graft landing above the celiac trunk [11,12]. This is especially useful in large-diameter false lumens and its successful use has also been reported in ruptured type B aortic dissection [13,14,15].

A second-generation device with a self-closing channel construction inside the plug and without the characteristic candy wrapper shape (an unsupported channel inside a standard tubular graft collapses and closes as soon as the dilator tip is removed) reported results in fourteen patients with 86% immediate complete false lumen occlusion; two required reintervention [16]. The authors concluded that the new candy-plug design reduces the procedural steps and offers good seal with minimal morbidity and mortality and a high rate of aortic remodeling [17].

In principle, the candy-plug will be compressed in the false lumen, but the possibility exists of compressing the true lumen instead with the risk of thrombosis of branch vessels located near the distal entries. Another concern is flap or vessel wall injury due to continuous shear stress by the device [18]. To address these concerns, a modified technique places a stent graft in the true lumen at the distal end of the occluding device in the false lumen [19,20].

## 2. Materials and Methods

This is a single-center, retrospective analysis of all consecutive patients with aortic dissection treated with false lumen embolization either with a vascular plug or with the candy-plug technique at Mainz University Medical Center between January 2017 and January 2022. All patients presented with acute, subacute (15–90 days), and chronic (>90 days) type A or B aortic dissections (TAAD, TBAD). The indications for false lumen embolization were as follows:

1. False lumen aneurysm > 5.5 cm.
2. Rapid aortic expansion (increase in aortic diameter > 5 mm in 6 months or > 10 mm in 1 year, especially if due to false lumen growth).
3. False lumen rupture (emergency indication).

High-resolution multi-slice computed tomography angiography (CTA) confirmed the diagnosis. Multiplanar and 3D workplace reconstructions were used to assess aortic lesions. CTA is the basis for case planning before discharge and standard-of-care follow-up at three and six months, and annually thereafter. Image evaluation was performed using 3Mensio Vascular software version 10.4 (3Mensio Medical Imaging B.V., Bilthoven, The Netherlands).

### 2.1. Endpoints

**Figure 1:**
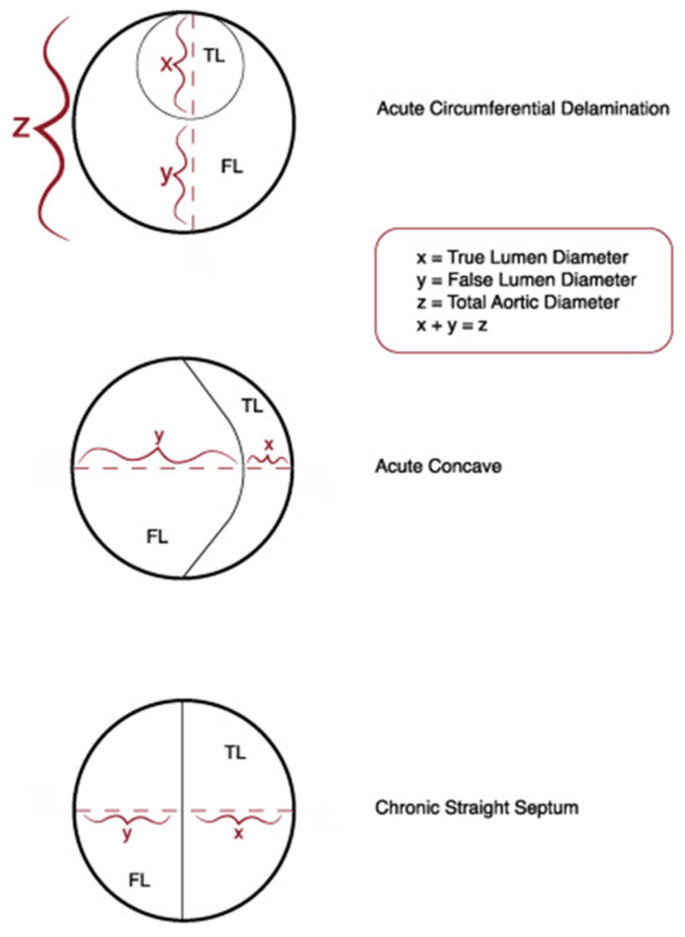
Diameter measurements of the aorta.

The primary endpoint was aortic remodeling, defined as true lumen augmentation and false lumen reduction. Measurements of the descending thoracic aorta (DTA) were obtained at the largest point between the left subclavian artery (LSA) and the celiac trunk before the procedure and the follow-up measurements were taken at the same level. These included the true lumen diameter (X), false lumen diameter (Y), and the largest horizontal total DTA diameter (Z), which were analyzed according to Society for Vascular Surgery and Society of Thoracic Surgeons reporting standards (Figure 1) [21].

Every attempt was made to obtain a true diameter measurement, and aortic angulation was considered when determining the location for each measurement; however, software that allows for automatic centerline measurements was not used. Each individual measurement was not necessarily taken at the same level in the aorta so that addition of the maximum true and false lumen diameters could result in a number that exceeds the largest total aortic diameter measured.

Secondary endpoints were as follows: technical success (defined as successful vascular plug or candy-plug delivery and deployment at the intended landing zone in the false lumen); and clinical success (defined as avoidance of perfusion at the site of occlusion on the final angiogram). In addition to diameter evaluation, the false lumen along the treated segment of aorta was evaluated for the presence of thrombosis and absence of retrograde flow from the descending aorta.

Other endpoints were as follows: neurological deficit, myocardial infarction, aortic rupture, neurological deficit, paraplegia, respiratory insufficiency (defined as tracheostomy, ventilation > 72 h, or reintubation), renal failure (permanent dialysis), organ failure, access or wound complications requiring secondary intervention. Other performance endpoints included the incidence of complications such as graft migration, erosion, infection, endovascular or open surgical secondary interventions, and all-cause and aortic-related mortality during the follow-up.

### 2.2. Candy-Plug: Device Description and Endovascular Surgical Procedure

**Figure 2:**
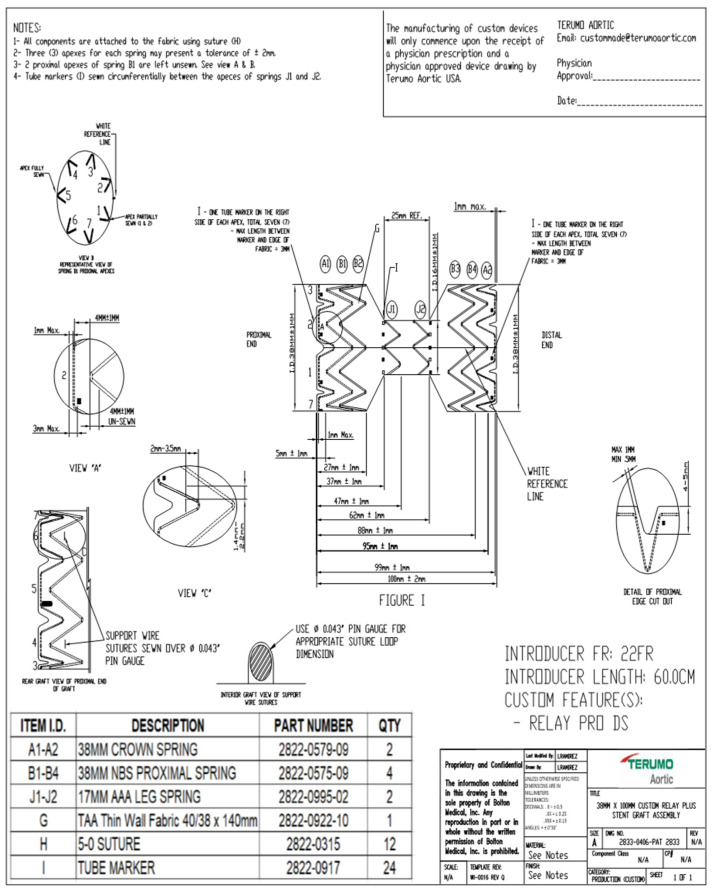
Candy-plug design.

A traditional candy-plug configuration is a stent graft with a narrow middle section (severe reduction in diameter towards the middle of the graft). We used two different candy-plug devices. The Cook Medical Candy-Plug and Iliac ZIP Occluder device have been described previously [12,17]. The Relay Candy-Plug (Terumo Aortic, Sunrise, FL, USA) is based on the standard RelayPlus or RelayPro NBS devices and comprises a self-expandable nitinol stent sutured to a polyester graft fabric [22], also with a series of sinusoidal stents with different diameters according to the position along the length of the stent graft (minimum length 70–75 mm; extra length might be necessary depending on the severity of the transition). The narrow middle section must have a minimum length of 10 mm and a minimum diameter of 14 mm and it is here that the vascular plug (Amplatzer, Abbott Cardiovascular, Chicago, IL, USA) is placed to embolize the false lumen. The diameters of the proximal and distal end of the candy-plug can be up to 50 mm (if based on RelayPlus; 44 mm if RelayPro) (Terumo Aortic, Sunrise, FL, USA). A minimum oversizing of 20% is anticipated (Figure 2).

Double vascular access with direct surgical femoral exposure allows a stiff wire to be placed in the ascending aorta through the true lumen, and a second one in the false lumen through distal re-entry. After standard TEVAR deployment in the true lumen and ballooning, the candy-plug device is deployed into the false lumen, taking care to ensure that the distal end of the candy-plug is 1–2 cm above the celiac trunk. After removal of the delivery system, a long sheath is inserted into the false lumen, ending above the narrow segment of the candy-plug and occluded with a vascular plug. In select patients with long- and narrow-diameter false lumens, it is possible to use a vascular plug alone (no candy-plug).

Statistical computations were performed using SPSS 22.0 for MAC (SPSS, Chicago, IL, USA). All frequency data are presented as percentages and all continuous data are presented as mean ± standard deviation: data were tested for normality and presented according to distribution. The confidence interval is 95%.

## 3. Results

A total of 12 dissection patients (83% type A, 17% type B; 33% with frank rupture) underwent TEVAR in conjunction with 13 false lumen embolization procedures. The patient demographics and characteristics are shown in Table 1. A high proportion had undergone prior aortic interventions: 83% underwent cardiac surgery, 83% underwent ascending aorta replacement, 67% underwent a prior TEVAR, and 25% underwent frozen elephant trunk (FET) arch replacement. Almost one-third of procedures were emergent (31%).

Cerebrospinal fluid (CSF) drainage was used prophylactically in two cases (15%) because many of the spinal arteries were perfused from the false lumen (Table 2). False lumen embolization was achieved with candy-plug stent grafts in 77% and vascular plug alone in 23%. The median operating time was 220 (100–300) minutes, and the median fluoroscopy time was 29.27 (14.04–49.17) minutes. The patients spent a median of 8 days (range, 4–27) in hospital.

One patient received a second vascular plug due to the migration of the first plug proximally above the narrow middle section. Intraoperative digital subtraction angiography showed the complete occlusion and absence of the false lumen perfusion above the plug. The technical success rate was 92% and the clinical success rate was 100% (Table 3).

There were no other intraoperative complications and no intraoperative mortality. There was no myocardial ischemia or surgical site infection. One patient had a transient ischemia attack. One patient had a secondary intervention within 30 days: the removal of a thoracic hematoma in the postoperative period for aortic rupture and worsened pulmonary function. One patient had two candy-plug procedures. This patient received post-TAAD ascending aorta surgical repair with good remodeling in the arch and residual dissection in the DTA, which had become aneurysmal and displayed expansion of the false lumen in the proximal thoracic aorta. We implanted a TEVAR in zone 2 in the true lumen and a candy-plug in the middle of the thorax in the false lumen, firstly, to minimize the risk of occluding an additional spinal artery over a long distance, and secondly, because we had a suitable landing zone in the mid-thoracic aortic segment. Over the course of about 10 months, the patient developed severe progressive dilation of the false lumen in the remaining part of the false lumen in the descending aorta distal to the implanted candy-plug, so it was decided to perform a TEVAR extension in the distal true lumen and a second candy-plug in the false lumen above the celiac trunk

Two patients (15%) died within 30 days. One patient had a history of TAAD, which was classified as postoperative respiratory decompensation due to severe chronic obstructive pulmonary disease (COPD) after a left pneumonectomy for carcinoma six years previously. The second patient died due to multiple organ failure due to alcohol abuse and cachexia.

The median follow-up time was 31 (3–76) months. Three further patients died during the follow-up period; none of these deaths were related to surgery. One patient died of cardiac decompensation by cardiomyopathy and low cardiac output; one due to sick sinus syndrome; and one of advanced ovarian cancer.

At follow-up, we noted on CT a 0.5 cm migration of the implanted candy-plug in one patient, which occurred approximately 12 months after implantation, but with no evidence of false lumen perfusion.

**Figure 3:**
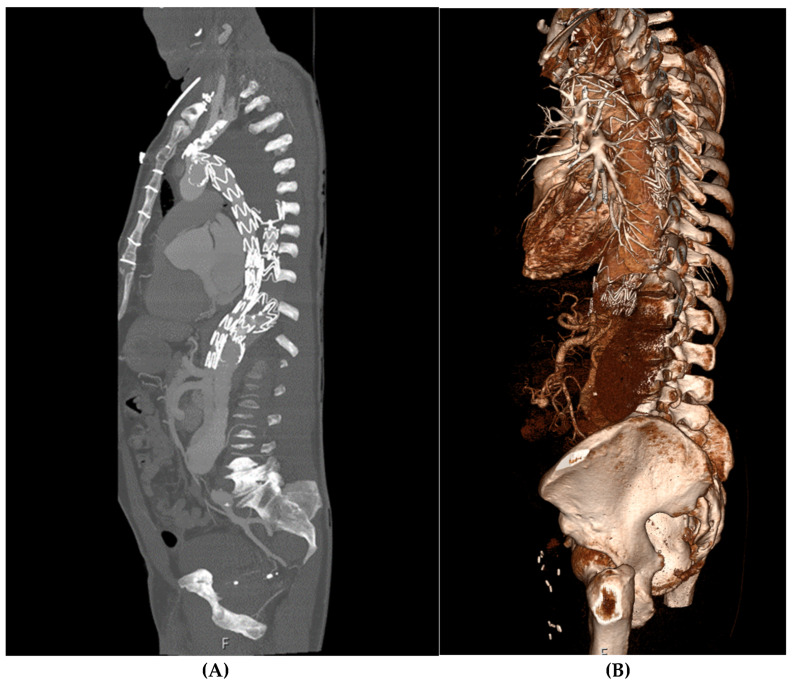
Postoperative computed tomography angiography (**A**) and three-dimensional rendering (**B**) of a patient treated with double candy-plug.

All of the patients remained free of symptoms, and all displayed positive aortic remodeling with complete false lumen thrombosis confirmed by CTA during follow-up. Two patients required a secondary intervention: one debranching in the aortic arch; a second underwent closure of the remaining thoracic false lumen above the celiac artery with candy-plug implantation with good outcomes (as described above) (Figure 3).

**Figure 4:**
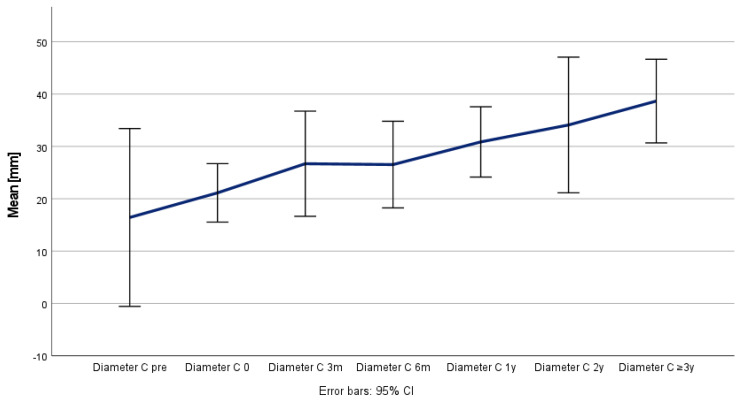
Remodeling and development of aortic true lumen (X) over time.

**Figure 5:**
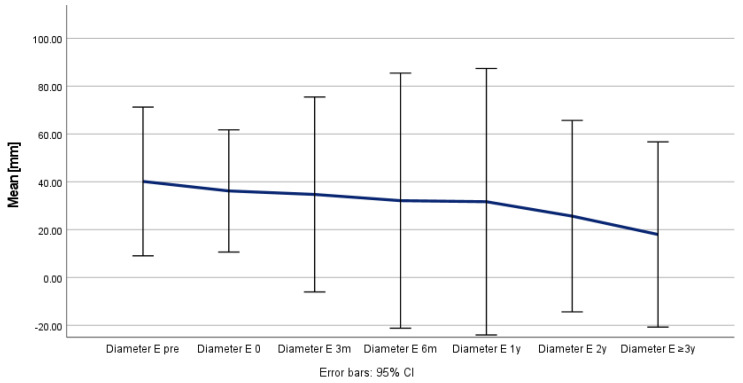
Remodeling and development of aortic false lumen (Y) over time.

**Figure 6:**
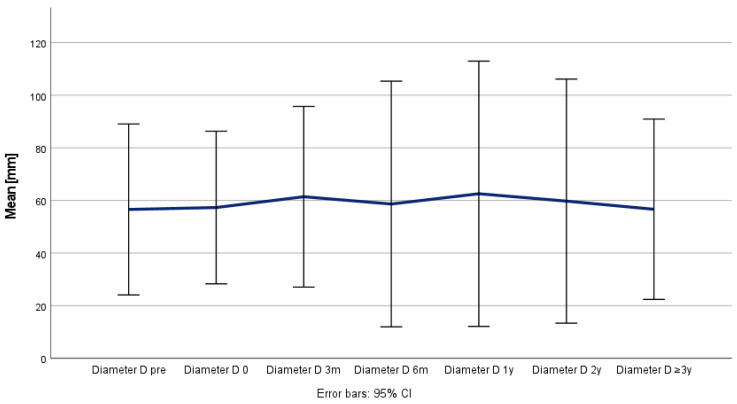
Remodeling and development of total aortic diameter (Z) over time.

**Figure 7:**
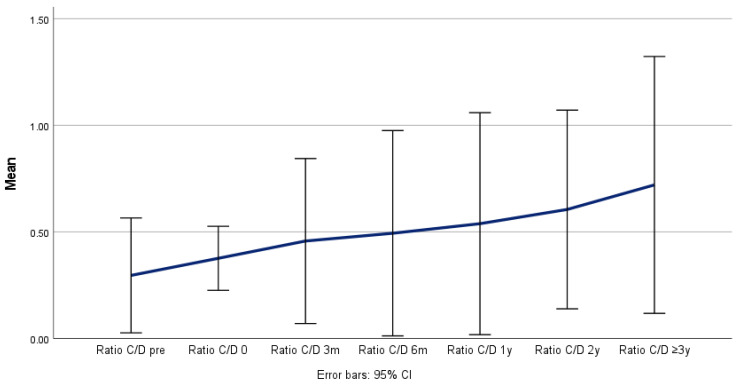
Remodeling and ratio of true lumen to total aortic diameter (X/Z) over time.

**Figure 8:**
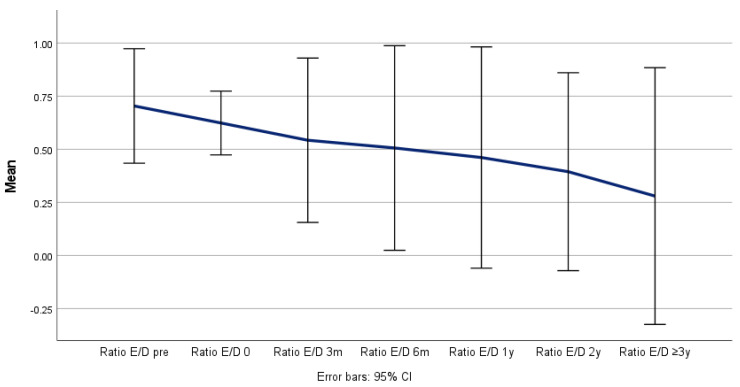
Remodeling and ratio of false lumen to total aortic diameter (Y/Z) over time.

Positive aortic remodeling was observed in all the patients, although the maximum thoracic aortic diameter (Z) remained stable (*p* = 0.526), as the true lumen (X) increased from 22 to 33 mm (*p* = 0.009) and the false lumen (Y) decreased from 41 to 20 mm (*p* = 0.076) during the follow-up period (Table 4 and Figure 4, Figure 5 and Figure 6) and was confirmed in the corresponding ratio results between the true lumen, false lumen, and total aortic diameter (Table 5 and Figure 7 and Figure 8).

## 4. Discussion

Over the years, since the first implantation by Dake in Massachusetts in 1990, TEVAR has established itself as the standard of care or therapy of choice for the thoracic aorta according to guidelines, and in particular for aortic dissection.

The primary goal of TEVAR, other than covering the entry tear or re-entry, is to restore antegrade blood flow in the true lumen and eliminate antegrade flow in the false lumen, thereby causing the re-expansion of the collapsed or narrowed true lumen and the obliteration or minimization of the false lumen, leading to positive aortic remodeling, which has been shown to have better results in terms of long-term survival [23,24,25,26]. Any false lumen perfusion has been associated with poorer outcomes compared to those in patients without [26]. Partial thrombosis in any aortic segment of a residual false lumen after aortic dissection repair correlates with a faster rate of aortic dilatation and predicts a higher rate of reoperation and, in the worst case, aortic rupture [23,27,28,29,30].

In addition, even with TEVAR, up to 20–40% of patients do not achieve false lumen obliteration because of the presence of retrograde entry flow. The volume and height of the flow is dependent on the number, size, and distance of the re-entries from the non-stented part of the thoracoabdominal aorta. The number of lumbar vessels originating from the false lumen also plays a role; Liu et al. found that cumulative thoracic aortic enlargement along the stent graft rate was higher in patients with eight or more preoperative false lumen branches compared to those with fewer than eight [8]. Intercostal arteries arising from the false lumen may also be a contributor to thrombosis inhibition [30].

Therefore, in addition to stenting the aortic true lumen, there is a need to obliterate false lumen perfusion to improve long-term results; several endovascular techniques have been described to achieve this, such as downstream stenting of the thoracoabdominal aorta, the knickerbocker technique, coils, plugs, intercostal embolization, glue, and candy-plugs [11,30,31,32,33,34].

Since the introduction of false lumen occlusion with an occlusion device, described as a “cork in the bottleneck”, by Loubert et al. in 2003 [35], Tilo Kölbel in Hamburg, Germany, has pioneered the candy-plug technique by adjusting a suture to limit the diameter from 42 to 10 mm in the middle part of the Zenith device (Cook Medical, Bloomington, IN, USA). This custom-made design was implanted in the false lumen mid-thoracic aorta and a 20 mm Amplatzer was implanted in the constricted part to achieve complete occlusion [12]. The technique was refined and updated outcomes were published by the same group in 2017 with custom-made devices with a restriction of 18 mm in the center, closed with a 22 mm vascular plug (St Jude) or 20 mm iliac zip occluder (Cook Medical). It was emphasized that the implantation of the candy-plug should be proximal to the celiac trunk, accompanied by a TEVAR in the true lumen deployed at the level of the stent graft in the true lumen to prevent stent-induced intimal tear. The rate of technical success was 100% and rate of clinical success was 94% in 18 patients with a mean follow-up of 9 months and aortic remodeling in 7/18 patients [11].

In 2019, the same group described a second-generation candy-plug (CPII) with a 22-F delivery system that was available with a diameter up to 46 mm and a length up to 71 mm. The CPII is tubular with no diameter restriction in the middle section and has a central tissue and channel within the graft. The central tissue channel collapses and prevents perfusion of the proximal false lumen and, therefore, no additional plug is required. The technical success rate was 100% and the clinical success rate was 93% in 18 patients, with a mean follow-up of 8 months. There was aortic remodeling in 8/10 patients (80%). The mean stay in intensive care was 1.4 ± 1 days.

The largest paper to date analyzing the real-world experience of the candy-plug technique (18 centers with 155 patients) showed a 100% technical success rate and positive aortic remodeling in 44% (68/155) at a median follow-up of 23 months [36]. A systematic review of patients after TAAD (n = 40) and chronic TBAD (n = 61) showed a technical success rate of 100% and 99% and a 30-day mortality of 2.5% and 0%, respectively, and false lumen thrombosis in 78% and 62%, respectively [37].

The limitation of the candy-plug concept is the size of the plug diameter (Cook up to 46 mm and Terumo up to 50 mm) and the availability in emergency cases of these custom-made stents [12,37]. An off-the-shelf candy-plug would be helpful in emergency cases of aortic false lumen rupture, which is why we have a candy-plug size of 40 mm with a length of 100 mm in our center ready for emergency cases: this was the case for four patients (31%) with false lumen aortic rupture in this series who were treated emergently. To address this issue in future studies, researchers might consider focusing on the following:

- Evaluating a wider range of standardized, commercially available candy-plug designs.
- Conducting simulation-based design studies.

This report is a single-center experience and there is a potential bias due to the small number of patients involved. Our follow-up period was limited to 31 (3–76) months. In our series, false lumen thrombosis was achieved with 100% technical and clinical success rates and positive aortic remodeling occurred during the follow-up period.

## 5. Conclusions

False lumen embolization appears to be safe and promotes false lumen thrombosis, which has a positive effect on aortic remodeling and favorable outcomes. An off-the-shelf candy-plug would be helpful in emergency cases of aortic false lumen rupture.

## Figures and Tables

**Table 1:** Patient and pathology characteristics.

	Mean ± SD	Median (Range) or n (%)
Male		11 (92)
Female		1 (8)
Age (years)	65.83 ± 11.7	63.0 (51–82)
Height (cm)	176.58 ± 5.37	178.5 (167–185)
Weight (kg)	83.42 ± 17.85	80.0 (55–115)
BMI (kg/m^2^)	26.63 ± 4.81	25.5 (20–36)
**Comorbidities**		
Hypertension		11 (92)
Hypercholesterolemia		9 (75)
Cigarette smoking		8 (67)
Active smoker		2 (17)
Former smoker		6 (50)
Chronic kidney disease		3 (25)
Dialysis		0 (0)
Prior stroke		3 (25)
Heart failure		3 (25)
Coronary stent		2 (17)
Coronary artery disease		2 (17)
Allergy		2 (17)
CABG		1 (8)
COPD		1 (8)
PAD		1 (8)
Diabetes mellitus		1 (8)
Myocardial infarction		0 (0)
Marfan syndrome		0 (0)
**Concomitant medication**		
Antihypertensive		12 (100)
Statin		8 (67)
Thrombocyte aggregation inhibitors		9 (75)
**Previous aortic interventions**		
Cardiac surgery		10 (83)
Ascending aorta replacement		10 (83)
Frozen elephant trunk		3 (25)
TEVAR		8 (67)
Supra-aortic trunk debranching		2 (17)
**Aortic pathology**		
Type A aortic dissection		10 (83)
Type B aortic dissection		2 (17)
Aortic rupture		4 (33)
Emergent		4 (31)
Elective		9 (69)
**ASA**		
I		0 (0)
II		1 (8)
III		5 (42)
IV		6 (50)
Proximal aortic diameter (mm)	37.56 ± 4.45	38.0 (28.0–42.0)
Distal aortic diameter (mm)	37.11 ± 4.37	38.0 (28.0–42.0)
Treatment length (mm)	204.89 ± 34.84	204.0 (161.0–259.0)
Tapered		1 (11)

ASA, American Society of Anesthesiologists; CABG, coronary artery bypass graft; COPD, chronic obstructive pulmonary disease; PAD, peripheral arterial disease; TEVAR, thoracic endovascular aortic repair.

**Table 2:** Procedural details of 13 candy-plug false lumen embolization procedures in 12 patients.

	Mean ± SD	Median (Range) or n (%)
Intubation		13 (100)
CSF drainage		2 (15)
**Index TEVAR**		12 (92)
Units used	1.23 ± 0.6	1.0 (0.0–2.0)
**First TEVAR device**		
RELAY		11 (92)
ZENITH		1 (8)
Proximal diameter (mm)	39.33 ± 5.61	40.0 (28.0–46.0)
Distal diameter (mm)	37.0 ± 4.55	37.0 (28.0–42.0)
Length (mm)	189.83 ± 31.54	204. 0 (104.0–209.0)
Tapered		7 (58)
**Second TEVAR device**		5 (39)
RELAY		5 (100)
Proximal diameter (mm)	36.0 ± 5.89	36.0 (30.0–42.0)
Distal diameter (mm)	34.0 ± 6.32	33.0 (28.0–42.0)
Length (mm)	156.5 ±41.13	159.0 (104.0–204.0)
Number of other stent grafts	0.08 ± 0.28	0.0 (0.0–1.0)
**False lumen embolization technique**		
**Vascular plug alone**		3 (23)
Number of vascular plugs used	2.33 ± 1.53	2.0 (1.0–4.0)
Size of the vascular plugs (mm)	21.67 ± 0.58	22.0 (21.0–22.0)
**Candy-plug**		10 (77)
Number of vascular plugs used		1.0 (1.0–2.0)
Size of vascular plugs (mm)		22.0 (20.0–24.0)
Subclavian closure system		2 (15)
Subclavian stent graft		1 (8)
Rapid pacing		2 (15)
Operating time (min)	197.92 ± 63.1	220.0 (100–300)
Fluoroscopy time (min)	31.17 ± 12.94	29.27 (14.04–49.17)
Dose area product (cGy/cm^2^)	4315.25 ± 2631.95	3497.44 (1198.4–8100.0)
Amount of contrast used (mL)	236.92 ± 96.67	220.0 (302.0–422.0)
Hospitalization (days)	12.39 ± 8.0	8.0 (4.0–27.0)
Intensive care (days)	1.69 ± 2.72	0.0 (0.0–8.0)

**Table 3:** Clinical outcomes.

	**Mean ± SD**	**Median (Range) or n (%)**
**Technical success**		12 (92)
**Clinical success**		13 (100)
**Complications**		
30-day mortality		2 (15)
Myocardial infarct		0 (0)
Respiratory insufficiency		3 (23)
Renal failure (permanent dialysis)		2 (15)
Neurological deficit		1 (8)
Aortic rupture		0 (0)
Paraplegia		0 (0)
Organ failure		0 (0)
Access or wound complication		0 (0)
Migration		1 (8)
Erosion		0 (0)
Infection		0 (0)
Secondary intervention		2 (16)
Within 30 days		1 (8)
Double candy-plug		1 (8)
**Laboratory parameters**		
Preoperative creatinine	1.09 ± 0.46	0.97 (0.71–2.45)
Preoperative urea	21.1 ± 10.84	19.0 (9.0–50.0)
Postoperative maximum creatinine	1.45 ± 0.79	1.08 (0.8–3.07)
Creatinine at discharge	1.03 ± 0.42	0.89 (0.7–2.16)
Urea at discharge	18.11 ± 7.88	17.0 (6.0–31.0)
**Transfusion**		
Erythrocyte concentrate		5 (38.5)
Volume	3.39 ± 7.75	0.0 (0.0–28.0)
Thrombocyte concentrate		1 (8)
**Follow-up (months) (n = 10)**	34.75 ± 31.1	31.0 (3.0–76.0)
**Late mortality**		3 (30)
Aortic-related		0 (0)
Time until death (month)	16.0 ± 15.62	8.0 (6.0–34.0)

Secondary interventions: thoracic hematoma removal; debranching; distal TEVAR extension and candy-plug.

**Table 4:** Aortic remodeling: changes in true lumen diameters (X), false lumen diameters (Y), and maximum thoracic aortic diameters (Z).

Timepoint CT Measurement	True Lumen Diameter (X) (mm)		False Lumen Diameter (Y) (mm)		Maximum Thoracic Aortic Diameter (Z) (mm)	
	Mean ± SD	Median (Range)	Mean ± SD	Median (Range)	Mean ± SD	Median (Range)
Preoperative	22.16 ± 8.84	22.0 (10.0–33.1)	40.22 ± 21.15	40.9 (11.3–83.0)	62.38 ± 19.03	63.25 (40.9–103.0)
Index	26.52 ± 6.66	28.4 (14.1–35.9)	35.97 ± 18.18	39.4 (10.9–75.0)	62.49 ± 18.96	63.0 (42.4–105.0)
3 months	27.94 ± 6.48	28.8 (14.4–37.0)	30.89 ± 16.61	34.6 (4.5–50.3)	58.83 ± 14.1	61.8 (37.7–77.0)
6 months	28.78 ± 5.24	28.4 (22.8–35.5)	26.23 ± 21.14	20.5 (8.5–55.4)	55.0 ± 17.01	50.55 (40.7–78.2)
1 year	30.1 ± 9.28	32.4 (12.8–38.6)	26.13 ± 23.04	20.0 (4.7–55.1)	56.23 ± 16.69	53.15 (41.3–82.9)
2 years	32.2 ± 9.43	34.45 (14.7–40.0)	22.72 ± 19.39	20.55 (4.3–49.3)	54.92 ± 15.09	54.6 (39.3–75.9)
≥3 years	33.4 ± 11.63	38.0 (13.0–41.0)	20.6 ± 20.84	27.0 (0.0–49.0)	54.0 ± 13.44	62.0 (38.0–67.0)
*p*-value	0.009		0.076		0.526	

CT, computed tomography.

**Table 5:** Aortic remodeling: changes in ratios of true lumen diameter (X) and false lumen diameter (Y) to the maximum thoracic aortic diameter (Z).

Timepoint CT Measurement	Ratio True Lumen to Max Thoracic Diameter (X/Z)		Ratio False Lumen to Max Thoracic Diameter (Y/Z)	
	Mean ± SD	Median (Range)	Mean ± SD	Median (Range)
Preoperative	0.39 ± 0.19	0.38 (0.14–0.74)	0.61 ± 0.19	0.62 (0.26–0.86)
Index	0.45 ± 0.16	0.44 (0.23–0.75)	0.55 ± 0.16	0.56 (0.25–0.77)
3 months	0.51 ± 0.2	0.52 (0.23–0.88)	0.49 ± 0.2	0.48 (0.12–0.77)
6 months	0.57 ± 0.22	0.6 (0.29–0.81)	0.43 ± 0.22	0.41 (0.19–0.71)
1 year	0.6 ± 0.29	0.64 (0.2–0.89)	0.4 ± 0.29	0.36 (0.11–0.8)
2 years	0.64 ± 0.27	0.67 (0.23–0.9)	0.36 ± 0.27	0.33 (0.1–0.77)
≥3 years	0.67 ± 0.33	0.6 (0.21–1.0)	0.33 ± 0.33	0.4 (0.0–0.79)
*p*-value	0.015		0.015	

CT, computed tomography.

## Data Availability

The data presented in this study are available on request from the corresponding author.

## Abbreviations

The following abbreviations are used in this manuscript:

CSF	Cerebrospinal fluid
CTA	Computed tomography angiography
DOAJ	Directory of Open Access Journals
DTA	Descending thoracic aorta
FET	Frozen elephant trunk
LSA	Left subclavian artery
MDPI	Multidisciplinary Digital Publishing Institute
NBS	Non-bare stent
TAAD	Type A aortic dissection
TBAD	Type B aortic dissection
TEVAR	Thoracic endovascular aortic repair

## References

[1] Ahlsson A., Wickbom A., Geirsson A., Franco-Cereceda A., Ahmad K., Gunn J., Hansson E.C., Hjortdal V., Jarvela K., Jeppsson A. (2019). Is There a Weekend Effect in Surgery for Type A Dissection?: Results From the Nordic Consortium for Acute Type A Aortic Dissection Database. Ann. Thorac. Surg..

[2] Pacini D., Di Marco L., Fortuna D., Belotti L.M.B., Gabbieri D., Zussa C., Pigini F., Contini A., Barattoni M.C., De Palma R. (2013). Acute aortic dissection: Epidemiology and outcomes. Int. J. Cardiol..

[3] Dohle D.-S., El Beyrouti H., Brendel L., Pfeiffer P., El-Mehsen M., Vahl C.-F. (2019). Survival and reinterventions after isolated proximal aortic repair in acute type A aortic dissection. Interact. Cardiovasc. Thorac. Surg..

[4] Ali-Hasan-Al-Saegh S., Halloum N., Scali S., Kriege M., Abualia M., Stamenovic D., Bashar Izzat M., Bohan P., Kloeckner R., Oezkur M. (2023). A systematic review and meta-analysis of retrograde type A aortic dissection after thoracic endovascular aortic repair in patients with type B aortic dissection. Medicine.

[5] Czerny M., Grabenwöger M., Berger T., Aboyans V., Della Corte A., Chen E.P., Desai N.D., Dumfarth J., Elefteriades J.A., Etz C.D. (2024). EACTS/STS Guidelines for diagnosing and treating acute and chronic syndromes of the aortic organ. Eur. J. Cardio Thorac. Surg. Off. J. Eur. Assoc. Cardio Thorac. Surg..

[6] Mani K., Clough R.E., Lyons O.T.A., Bell R.E., Carrell T.W., Zayed H.A., Waltham M., Taylor P.R. (2012). Predictors of Outcome after Endovascular Repair for Chronic Type B Dissection. Eur. J. Vasc. Endovasc. Surg..

[7] Roselli E.E. (2015). Thoracic endovascular aortic repair versus open surgery for type-B chronic dissection. J. Thorac. Cardiovasc. Surg..

[8] Liu F., Ge Y.Y., Guo W., Liu X.P., Jia X., Xiong J., Ma X.H. (2018). Preoperative thoracic false lumen branches are predictors of aortic enlargement after stent grafting for DeBakey IIIb aortic dissection. J. Thorac. Cardiovasc. Surg..

[9] Lescan M., Veseli K., Oikonomou A., Walker T., Lausberg H., Blumenstock G., Bamberg F., Schlensak C., Krüger T. (2017). Aortic Elongation and Stanford B Dissection: The Tübingen Aortic Pathoanatomy (TAIPAN) Project. Eur. J. Vasc. Endovasc. Surg..

[10] Hosseini M., Blitzer D.N., Ghazi A., Toursavadkohi S. (2020). Flow Diversion: A Novel Technique In The Management Of Aneurysmal False Lumen In Chronic Type B Aortic Dissection. Ann. Vasc. Surg..

[11] Rohlffs F., Tsilimparis N., Fiorucci B., Heidemann F., Debus E.S., Kölbel T. (2017). The Candy-Plug Technique: Technical Aspects and Early Results of a New Endovascular Method for False Lumen Occlusion in Chronic Aortic Dissection. J. Endovasc. Ther..

[12] Kölbel T., Lohrenz C., Kieback A., Diener H., Debus E.S., Larena-Avellaneda A. (2013). Distal False Lumen Occlusion in Aortic Dissection with a Homemade Extra-Large Vascular Plug: The Candy-Plug Technique. J. Endovasc. Ther..

[13] Marone E.M., Leopardi M., Bertoglio L., Mascia D., Chiesa R. (2017). Original Off-Label Endovascular Solution to Occlude False Lumen Rupture in Chronic Type B Aortic Dissection. Ann. Vasc. Surg..

[14] Lin C.-Y., Su I.-H., Chu S.-Y., Ko P.-J. (2018). Modified Candy-Plug Technique for Rescue Type B Aortic Dissection with False-Lumen Rupture. Ann. Vasc. Surg..

[15] Morisaki A., Sohgawa E., Kishimoto N., Yamane K., Shibata T. (2019). Candy-plug technique for ruptured chronic type B aortic dissection. Asian Cardiovasc. Thorac. Ann..

[16] Rohlffs F., Tsilimparis N., Mogensen J., Makaloski V., Debus S., Kölbel T. (2019). False lumen occlusion in chronic aortic dissection: The new generation Candy-Plug II. Ann. Vasc. Surg..

[17] Eleshra A., Kölbel T., Tsilimparis N., Panuccio G., Scheerbaum M., Debus E.S., Mogensen J., Rohlffs F. (2019). Candy-Plug Generation II for False Lumen Occlusion in Chronic Aortic Dissection: Feasibility and Early Results. J. Endovasc. Ther..

[18] Ogawa Y., Nishimaki H., Chiba K., Murakami K., Sakurai Y., Fujiwara K., Miyairi T., Nakajima Y. (2016). Candy-Plug Technique Using an Excluder Aortic Extender for Distal Occlusion of a Large False Lumen Aneurysm in Chronic Aortic Dissection. J. Endovasc. Ther..

[19] Kotani S., Inoue Y., Kasai M., Suzuki S., Hachiya T. (2017). Modified ‘candy-plug’ technique for chronic type B aortic dissection with aneurysmal dilatation: A case report. J. Cardiothorac. Surg..

[20] Furukawa T., Uchida N., Yamane Y., Yamada K. (2017). A pitfall of false lumen embolization in chronic aortic dissection: Intimal injury caused by the embolization device edge. Interact. Cardiovasc. Thorac. Surg..

[21] Lombardi J.V., Hughes G.C., Appoo J.J., Bavaria J.E., Beck A.W., Cambria R.P., Charlton-Ouw K., Eslami M.H., Kim K.M., Leshnower B.G. (2020). Society for Vascular Surgery (SVS) and Society of Thoracic Surgeons (STS) reporting standards for type B aortic dissections. J. Vasc. Surg..

[22] Beyrouti H.E., Lescan M., Doemland M., Mustafi M., Jungmann F., Jorg T., Halloum N., Dorweiler B. (2020). Early results of a low-profile stent-graft for thoracic endovascular aortic repair. PLoS ONE.

[23] Tsai M.-T., Wu H.-Y., Roan J.-N., Tsai Y.-S., Hsieh P.C.H., Yang Y.-J., Luo C.-Y. (2014). Effect of false lumen partial thrombosis on repaired acute type A aortic dissection. J. Thorac. Cardiovasc. Surg..

[24] Bernard Y., Zimmermann H., Chocron S., Litzler J.F., Kastler B., Etievent J.P., Meneveau N., Schiele F., Bassand J.P. (2001). False lumen patency as a predictor of late outcome in aortic dissection. Am. J. Cardiol..

[25] Elefteriades J.A., Lovoulos C.J., Coady M.A., Tellides G., Kopf G.S., Rizzo J.A. (1999). Management of descending aortic dissection. Ann. Thorac. Surg..

[26] Sueyoshi E., Sakamoto I., Uetani M. (2009). Growth rate of affected aorta in patients with type B partially closed aortic dissection. Ann. Thorac. Surg..

[27] Trimarchi S., Tolenaar J.L., Jonker F.H.W., Murray B., Tsai T.T., Eagle K.A., Rampoldi V., Verhagen H.J.M., van Herwaarden J.A., Moll F.L. (2013). Importance of false lumen thrombosis in type B aortic dissection prognosis. J. Thorac. Cardiovasc. Surg..

[28] Tolenaar J.L., Eagle K.A., Jonker F.H.W., Moll F.L., Elefteriades J.A., Trimarchi S. (2014). Partial thrombosis of the false lumen influences aortic growth in type B dissection. Ann. Cardiothorac. Surg..

[29] Tsai T.T., Evangelista A., Nienaber C.A., Myrmel T., Meinhardt G., Cooper J.V., Smith D.E., Suzuki T., Fattori R., Llovet A. (2007). Partial thrombosis of the false lumen in patients with acute type B aortic dissection. N. Engl. J. Med..

[30] Magee G.A., Yi J.A., Kuwayama D.P. (2020). Intercostal artery embolization to induce false lumen thrombosis in type B aortic dissection. J. Vasc. Surg. Cases Innov. Tech..

[31] Oderich G.S., Ribeiro M., Reis de Souza L., Hofer J., Wigham J., Cha S. (2017). Endovascular repair of thoracoabdominal aortic aneurysms using fenestrated and branched endografts. J. Thorac. Cardiovasc. Surg..

[32] Hofferberth S.C., Nixon I.K., Mossop P.J. (2012). Aortic false lumen thrombosis induction by embolotherapy (AFTER) following endovascular repair of aortic dissection. J. Endovasc. Ther. Off. J. Int. Soc. Endovasc. Spec..

[33] Nienaber C.A., Kische S., Zeller T., Rehders T.C., Schneider H., Lorenzen B., Bünger C., Ince H. (2006). Provisional extension to induce complete attachment after stent-graft placement in type B aortic dissection: The PETTICOAT concept. J. Endovasc. Ther. Off. J. Int. Soc. Endovasc. Spec..

[34] Roselli E.E., Idrees J., Reside J., Shafii S. (2014). Use of Covered Stent Devices for False Lumen Embolization in Chronic Dissection: A Novel Approach. Ann. Thorac. Surg..

[35] Loubert M.C., van der Hulst V.P.M., De Vries C., Bloemendaal K., Vahl A.C. (2003). How to Exclude the Dilated False Lumen in Patients after a Type B Aortic Dissection? The Cork in the Bottleneck. J. Endovasc. Ther..

[36] Eleshra A., Haulon S., Bertoglio L., Lindsay T., Rohlffs F., Dias N., Tsilimparis N., Panuccio G., Kölbel T., Candy-Plug Collaborators (2023). Custom Made Candy Plug for Distal False Lumen Occlusion in Aortic Dissection: International Experience. Eur. J. Vasc. Endovasc. Surg. Off. J. Eur. Soc. Vasc. Surg..

[37] Spanos K., Kölbel T., Rohlffs F., Heidemann F., Giannoukas A.D., Debus S.E., Tsilimparis N. (2019). Intentional Targeted False Lumen Occlusion after Aortic Dissection: A Systematic Review of the Literature. Ann. Vasc. Surg..

